# Dynamic ECG signal quality evaluation based on persistent homology and GoogLeNet method

**DOI:** 10.3389/fnins.2023.1153386

**Published:** 2023-03-08

**Authors:** Yonglian Ren, Feifei Liu, Shengxiang Xia, Shuhua Shi, Lei Chen, Ziyu Wang

**Affiliations:** ^1^School of Science, Shandong Jianzhu University, Jinan, China; ^2^Center for Engineering Computation and Software Development, Shandong Jianzhu University, Jinan, China; ^3^School of Science and Technology, Shandong University of Traditional Chinese Medicine, Jinan, China

**Keywords:** quality assessment, persistent homology, point cloud, complex sequence, persistent barcode

## Abstract

Cardiovascular disease is a serious health problem. Continuous Electrocardiograph (ECG) monitoring plays a vital role in the early detection of cardiovascular disease. As the Internet of Things technology continues to mature, wearable ECG signal monitors have been widely used. However, dynamic ECG signals are extremely susceptible to contamination. Therefore, it is necessary to evaluate the quality of wearable dynamic ECG signals. The topological data analysis method (TDA) with persistent homology, which can effectively capture the topological information of high-dimensional data space, has been widely studied. In this study, a brand-new quality assessment method of wearable dynamic ECG signals was proposed based on the TDA with persistent homology method. The point cloud of an ECG signal was constructed, and then the complex sequence was generated and displayed as a persistent barcode. Finally, GoogLeNet based on the transfer learning model with a 10-fold cross-validation method was used to train the classification model. A total of 12-leads ECGs Dataset and single-lead ECGs Dataset, established based on the 2011 PhysioNet/CinC challenge dataset, were both used to verify the performance of this method. In the study, 773 “acceptable” and 225 “unacceptable” signals were used as 12-leads ECGs Dataset. We relabeled 12,000 ECG signals in the challenge dataset, and treated them as single-lead ECGs Dataset after empty lead detection and balance datasets. Compared with the traditional ECG signal quality assessment method mainly based on waveform characteristics and time-frequency characteristics, the performance of the quality assessment method proposed. In this study, the classification performance of the proposed method are fairly great, *mAcc* = 98.04%, *F*1 = 98.40%, *Se* = 97.15%, *Sp* = 98.93% for 12-leads ECGs Dataset and *mAcc* = 98.55%, *F*1 = 98.62%, *Se* = 98.37%, *Sp* = 98.85% for single-lead ECGs Dataset.

## Introduction

Heart disease is a serious threat to human health. According to the World Health Report 2021, by 2019, cancer, cardiovascular disease (CVD), diabetes, and chronic respiratory disease will be the main killers of human beings. Therefore, the prevention, diagnosis, and treatment of CVD have become important issues worldwide. With the rapid development of networks, big data, the Internet of Things, and artificial intelligence, wearable ECG monitoring equipment (Redmond et al., 2012; Viegas et al., 2016) has been widely used. Realize real-time and long-term monitoring of human ECG signals. However, ECG signal is a weak physiological signal that is easy to be interfered, leading to obvious defects in recording signal quality. Therefore, it is necessary to conduct effective signal quality assessment when monitoring wearable ECG equipment.

There are the following research methods for ECG signal quality evaluation, which are based on PhysioNet/CinC competition data in 2011. Zaunseder et al. (2011) based on the frequency domain characteristics of ECG signals, used indicators related to the power spectrum combined with decision trees to classify ECG signals. The classification accuracy was 90.4%; Kalkstein et al. (2011) used a combined machine learning algorithm of K-nearest neighbor and random forest for ECG signal quality assessment and obtained 91.2% classification accuracy on the test set; Xia et al. (2011) studied the time domain, frequency domain, autocorrelation, cross-correlation, and other indicators of ECG time series, formed a matrix with the results of these indicators, and used the spectral radius of the regular matrix to classify ECG signals. With the rapid development of deep learning, researchers began to apply deep learning to cardiovascular disease classification. For example, Alqudah et al. (2022) sent the extracted ECG spectrum features to different convolutional neural network (CNN) architectures to classify the MIT-BIH arrhythmia database. Al-Issa and Alqudah (2022) developed a heart diagnostic system combining CNN and Long Short-Term Memory (LSTM) components to distinguish five heart valve diseases. Obeidat and Alqudah (2021) used a hybrid lightweight one-dimensional depth learning model, which combines convolutional CNN and LSTM methods for ECG classification.

Topological data analysis method is a data analysis framework based on algebraic topology tools (Carlsson, 2009). The purpose of adopting the TDA method is to apply data analysis, algebraic topology, computational geometry, computer science, statistics, etc., to find a shape like structure in the data to analyze the complex topology and geometry of the data (Edelsbrunner and Harer, 2010). These data are usually represented as a point cloud in Euclidean space. Persistent homology (Zomorodian and Carlsson, 2005) is the main concept that allows multiscale data analysis and is also a basic mathematical tool of TDA. Persistent homology is calculated by simple complex, and its output results usually include persistent barcode and persistent diagram. In this study, Vietoris-Rips (VR) complex and SubLevel-Set (SLS) complex are selected, and detailed in Section “2. Basic terminology.”

Topological data analysis method methods have been applied to wearable ECG signal analysis [see (Chung et al., 2021) for an example] based on the persistence diagram obtained by VR filtering and SLS filtering. This is used to construct persistence statistics for heart rate variability analysis and its classification in sleep-wake. Reference (Dindin et al., 2020) used the TDA method to detect arrhythmias through a modular multichannel neural network for binary classification. The classification accuracy obtained in the test set was 90% on average, and the average test accuracy in multiclassification was 80.5%. Reference (Ignacio et al., 2019) demonstrated how to map ECGs onto high-dimensional point clouds through delayed embedding to extract topological features and finally apply random forests for classification. Study (Graff et al., 2021) examined when persistence diagram was obtained by SLS filtering, and a set of indicators was extracted to distinguish the RR interval of healthy subjects and stroke patients. In addition (Yan et al., 2019) applied TDA to reconstruct a signal point cloud to extract persistent landscape features to classify heart rate variability. The accuracy of a normal heartbeat was 100%, of ventricular beating was 97.13%, of supraventricular beating was 94.27%, and of fusion beating was 94.27%. Although the TDA method has been applied to the processing and classification of ECG signals, to the best of our knowledge, research on the quality assessment of ECG signals using TDA is still lacking.

Traditional ECG signal quality evaluation methods mostly rely on the setting of ECG signal feature extraction classifier. In recent years, the deep learning method has been widely used in many fields because of its powerful functions. More and more researchers apply the deep learning method to the quality evaluation of ECG signals. However, the deep learning method has poor interpretability and cannot explore the high-dimensional spatial characteristics of ECG signals. ECG is an electrical activity process that reflects the excitation of the heart. It is not enough to extract features from the basic function of the heart and its pathological research. Topological data analysis method can solve this problem. In this study, persistent homology can be used to construct point clouds through folded signals, extract topological features, and comprehensively reflect the damage of heart valves.

In this study, a brand-new quality assessment method of wearable dynamic ECG signals was proposed based on the TDA with persistent homology method, so as to reduce the workload of medical staff and reduce the rate of miscarriage of justice. In this study, the features captured by the topological data analysis method is topological and spatial information of high-dimensional data space. First, the point cloud of an ECG signal was constructed, and then the complex sequence was generated and displayed as a persistent barcode. Topological and spatial information was converted into the persistent barcode.

In Section “2. Basic terminology,” we introduce VR filtration, SLS filtration, and persistent homology related concepts. The dataset adopted in this study and the constructed model are accepted in detail in Section “**3. Model**.” In Section “4. Results,” we present the results for different classifications. Model performance based on ECG quality assessment and comparison with other quality assessment methods is discussed in detail in Section “5. Discussion.”

## Basic terminology

### Vietoris-Rips filtration

**FIGURE 1:**
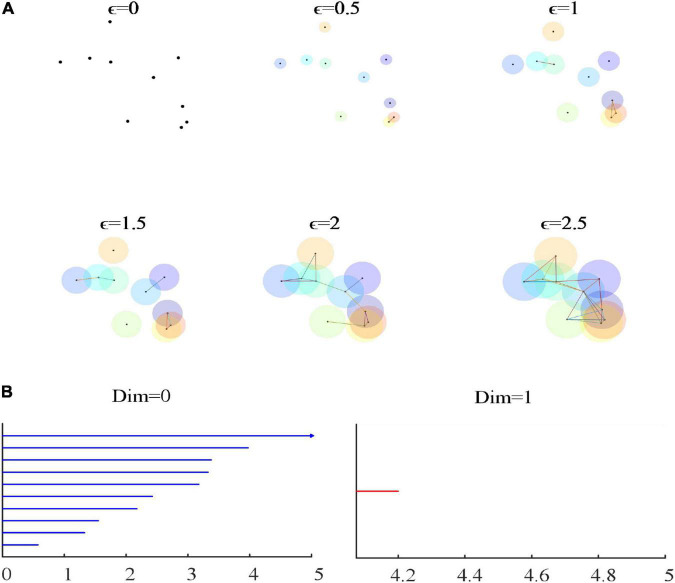
**(A)** The filtering process of the VR complex. **(B)** The 0-dimensional and 1-dimensional persistence barcode of VR filtration. As ∈ continues to increase, an increasing number of simplicial complexes are formed. In **(B)**, blue is a 0-dimensional persistence barcode, and red is a 1-dimensional persistence barcode. The abscissa of the persistence barcode picture represents 2∈. When ∈ = 0, there are 10 connected components; when ∈ = 1, there are seven connected components; when ∈ = 2, there are two connected branches; and when ∈ = 2.05 to ∈ = 2.1, there is a hole.

Common complexes include Alpha complex, $\hat{C}\,e\,c\,h$ complex, lazy witness complex, VR complex and so on. In this study, VR complex is selected for the following reasons: (a) when using Alpha complex, the lack of monotonicity may introduce significant computational costs and even make it unusable in some cases. (b) In the calculation, VR complex is easier to calculate than $\hat{C}\,e\,c\,h$ complex. Because VR complex can be stored as a picture, that is, only 0-dimensional and 1-dimensional complex need to be stored, and all high-dimensional complex need not be stored like $\hat{C}\,e\,c\,h$ complex. (c) The lazy witness complex is to randomly select the number of points in a group of point clouds. Compared with VR complex, it has randomness and is suitable for the point cloud structure with a large amount of data. Assume that VR filtering represents the distance between two points in the metric space Z. The VR complex sequence VR (Z, ∈) is defined as follows: (1) The vertex set is Z. (2) For vertices a and b, if d (a, b) ≤ ∈, then the edge (ab) is included in VR (Z, ∈). (3) If all edges of VR (Z, ∈) are simplexes, then it contains simplexes of higher dimensions. The filtering of the VR complex can be regarded as filtration of the (n-1) dimensional simplex, as shown in Figure 1.

### SubLevel-Set filtration

The definition of the SLS is as follows: We define a time series of ECG signals as a continuous function, where *T* is the length of the time series and is a real-valued function called the α-level subset. For each α∈R,


(1) $$f_{\alpha }:=f^{-1}\,\left(-\infty ,\alpha \right)\,\left\{\text{t}\in \left[0,T\right]|f\,\left(x\right)\leq \alpha \right\}$$

**FIGURE 2:**
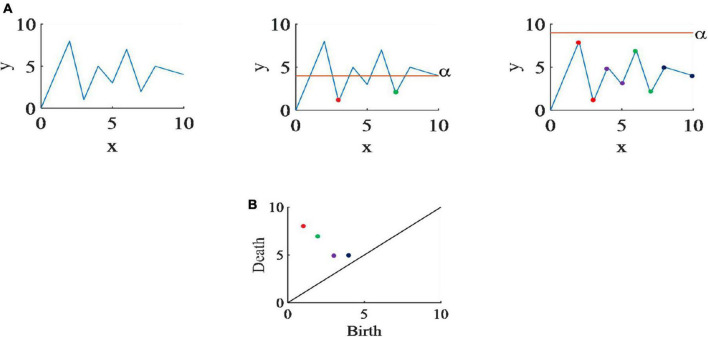
**(A)** The SLS filtering process of randomly generated time series. When α scans a node that it considers to be a local minimum, it saves the value of that node as the birth point of the slot, and the trough of death is determined by the lowest. Since the global minimum will not disappear, its death time is infinite. **(B)** The persistence diagram of this time series.

According to the formula α_1_ ≤ α_2_, *f*_α_1__ ⊆ *f*_α_2__, therefore, for any increasing sequence of a filter is formed. The filtering process is shown in Figure 2 below.

### Persistence homology

Persistent homology is a method to compute spatial topological features at different spatial resolutions. More persistent features are detected across a wide range of spatial scales. The space must first be represented as a simplicial complex, and a distance function on the underlying space corresponds to a filtering of a simplicial complex, which is a nested sequence of increasing subsets.

When 0≤*i*≤*j*≤*n*, the inclusio *K*_*i*_→*K*_*j*_ induces a homomorphism $f_{p}^{i,j}:H_{p}\,\left(K_{i}\right)\to H_{p}\,\left(K_{j}\right)$ on the simplicial homology groups for each dimension p. The *p^th^* persistent homology groups are the images of these homomorphisms, and the *p^th^* persistent Betti numbers *p* = 0 are the ranks of those groups. Persistent Betti numbers for coincide with the size function, which is a predecessor of persistent homology.

Topological data analysis method methods extract information from the topological and geometric properties of the data point cloud. In this study, we first construct a data point cloud for each time series using the sliding window method to construct a complex sequence for a point cloud dataset and filter the complex sequence ϕ = *K*_0_ ⊂ *K*_1_ ⊂ … ⊂ *K*_n_ = *K*. Topological features will appear and disappear during the construction of complex filtering. The persistence diagram proposed by Edelsbrunner et al. (2000) and the persistence barcode proposed by Carlsson et al. (2005) are tools for visualizing topological features that can visually display persistent homology. There is an equivalence relationship between them. The persistence diagram was encoded from the k-dimensional homology α information in all scales. A homology α was a point, which represent the birth and death time of the corresponding topological features. A barcode is a finite set of intervals that are bounded below. Intuitively, the intervals denote the life-times of a non-trivial loop in a growing complex. The left endpoint signifies the birth of a new topological attribute, and the right endpoint signals its death. The longer the interval, the more important the topological attribute, as it insists on being a feature of the complex.

## Model

**FIGURE 3:**
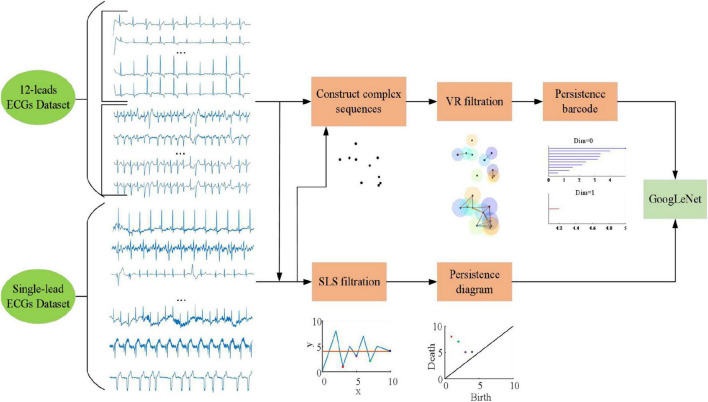
Flow diagram of this study.

Based on 2011 PhysioNet/CinC challenge data, we constructed 12-leads ECGs Database and single-lead ECGs Database. For the two databases, we use VR filtration method to obtain persistent barcodes and SLS filtration method to obtain persistent diagrams. When using VR filtration method, this study uses the sliding window method to construct the point cloud of the ECG signal. Finally, the GoogLeNet based on the transfer learning method is applied for classification. The flow diagram of this study is shown in Figure 3.

### The data

Data were drawn from the PhysioNet Challenge 2011 dataset where binary labels were available, on 1,000 12-lead ECGs indicating whether the entire recording was acceptable or unacceptable. These data supporting the development and evaluation of challenge entries were collected by the Sana Project (Celi et al., 2009) and are freely available through PhysioNet (Goldberger et al., 2000). Patient age, sex, weight, and possibly other relevant information were included in the challenge data. The full diagnostic bandwidth is 0.05–100 Hz. Leads were recorded simultaneously for 10 s, sampled at 500 Hz at 16-bit resolution. Among the 1,000 signals, 773 were marked as “acceptable” and 225 were “unacceptable,” and 2 were “indeterminate.”

#### A total of 12-leads ECGs dataset

In this study, 773 “acceptable” and 225 “unacceptable” signals were used as 12-leads ECGs Dataset. Whereas the “acceptable,” “indeterminate,” and “unacceptable” classification criteria for the entire 12 channels. For example, many “acceptable” ECGs have a channel with complete noise or even a flat line. Therefore, we constructed single-lead ECGs Dataset.

#### Single-lead ECGs quality assessment dataset construction

According to Silva et al. (2011), study (Liu et al., 2018) adopts the scoring criteria of five signal quality levels of 10-s ECG segments. A total of 9,941 “acceptable” and 2,059 “unacceptable” 10-s ECG segments were found. With empty lead detection, 1,071 10-s ECG segments were detected from the disqualified group. Hence, only 988 “unacceptable” fragments were found. It can be seen that the “acceptable” and “unacceptable” signals are seriously unbalanced, and this study generates additional noisy records to balance the problem of uneven data.

**FIGURE 4:**
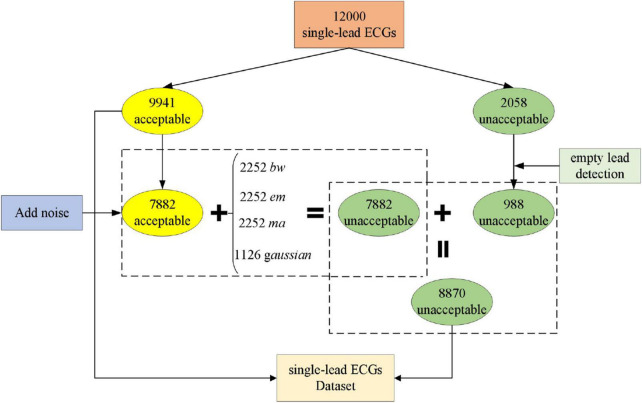
Construction process of single-lead ECGs Dataset.

We used the Physical Network Noise Stress Test Database (Moody et al., 1984) (NSTDB) noise samples, which contain samples for three types of noise: *bw*, *em*, and *ma*. *Bw* contains baseline drift noise; *em* contains electrode motion artifacts, as well as substantial baseline drift and muscle noise; and *ma* contains mostly muscle noise. These three noise samples have two leads. This study adds *gaussian* noise with only one lead data, and the signal-to-noise ratio of the noise is −10 dB. We added four different noises to the 7,882 “acceptable” ECG signals: 2,252 noise data with *bw*, *em*, and *ma* noise and 1,126 with *gaussian* noise. There is no possibility of adding two different noises to one signal. There are 9,941 “acceptable” signals and 8,870 “unacceptable” signals. As shown in the Figure 4.

#### Electrocardiograph signal processing

The ECG signals of “acceptable” and “unacceptable” were normalized by Mapminmax function. Mapminmax is a function of MATLAB, which is mainly used to normalize data. It converts all data into numbers between (−1, 1), so as to eliminate the difference in the number of data in each dimension. The algorithm is as follows:

It is assumed that *x* has only finite real values and that the elements of each row are not all equal. *ymin* is the minimum value we expect after normalization. *ymax* is the maximum value we expect after normalization, and the normalized matrix is marked as *y*.


(2) $$y=\frac{\left(y\,m\,a\,x-y\,m\,i\,n\right)\,\left(x-x\,m\,i\,n\right)}{\left(x\,m\,a\,x-x\,m\,i\,n\right)}+y\,m\,i\,n$$

### Filtration of ECG signal

**FIGURE 5:**
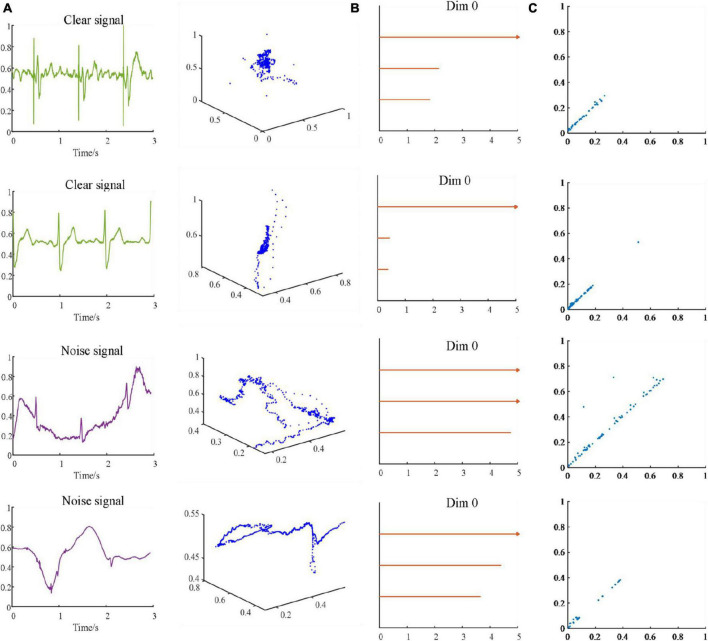
Two clean and two noisy ECG signals and its corresponding use of the sliding window method to establish three-dimensional scatter **(A)** and persistence barcode **(B)**. Persistence diagram obtained by the SLS-filtration method **(C)**.

Topological data analysis method studies shapes constructed from invariant datasets under continuous deformation (such as tension and torsion). We use VR filtration and SLS filtration methods to analyze ECG signals, and use persistence barcode and persistence diagram to display the topological characteristics of ECG signals. In VR filtration method, a point cloud was structure by the sliding window method. In the process of ∈ becoming larger, points are connected with each other, and “hole” and “void” may appear and disappear, which means that the topological characteristics will appear and disappear. SLS filtration method directly looks for birth value and death value on the waveform of ECG signal. In order to see the difference between persistent barcodes more intuitively and clearly, we take a 3-s ECG signal segment in Figure 5 as an example. Figure 5 shows the three-dimensional scatter (A), persistence barcode (B), and persistence diagram (C) of 3-s “acceptable” and “unacceptable” ECG segments.

In this study, for 12-leads ECGs Dataset, the point cloud structure of sliding windows with five lengths of 1, 2, 3, 4, and 5 s and dimensions of 120, 60, 36, 24, and 24 are established. For single-lead ECGs Dataset, the point cloud structure of sliding windows with five lengths of 0.1, 0.2, 0.3, 0.4, and 0.5 s and dimensions of 100, 50, 33, 25, and 20 are established. When a signal cannot meet the length of a sliding window, we discard it. For the 12-leads dataset, when the sliding window length is 0.1 s, the point cloud dimension of one signal is 120, and the point cloud dimension of 12-leads signals is 1,200. For a single-lead dataset, if the sliding window length is 1 s, the point cloud dimension is 10. According to the experimental results, we know that the larger the dimension of the point cloud, the better the result. However, when the point cloud dimension is too large, it will cause a certain amount of calculation and time loss. Therefore, in this study, we control the size of the sliding window to control the dimension of the two data sets within 120, which can not only maintain the accuracy of the results, but also reduce unnecessary waste of time.

#### Sliding window method to construct point cloud and VR filtration of ECG signal

At present, the application of ECG signal quality assessment based on persistent homology is lacking. In this study, the sliding window method is used to establish the point cloud dataset. Given a set of time series *x*(*x*_1_, *x*_2_, …, *x*_*n*_), construct a matrix,


(3) $$\begin{array}{c} \left[\begin{array}{c} x_{1},\ldots ,x_{t}\\ x_{t+1},\ldots ,x_{2\,t}\\ \ldots \\ x_{\left(d-1\right)\,t+1},\ldots ,x_{d\,t} \end{array}\right],d\,t\leq \text{n},d>0,t>0,n>0 \end{array}$$

Where *d* is the dimension, *t* is the size of the sliding window, and *n* is the length of the time series. At that time, a point that does not meet the size of a window is discarded. Through experiments, the window size is continuously adjusted to find an optimal window size so that the classification accuracy is the best.

The process of constructing VR complex is reconstruct the point cloud from the time series of ECG signals. Each point is surrounded by a ball with a diameter of 2∈. During the change of radius ∈, holes will appear and disappear. The following is an example of intercepting a 3-s ECG signal to reconstruct the three-dimensional scatter of the point cloud dataset to better visualize the spatial structure of the point cloud. As shown in Figures 5A, B, the length of the sliding window is 1 s, and the dimension is three.

#### SubLevel-Set filtration of ECG signal

SubLevel-Set filtering method maps time series data to its peak and trough pairs to express information about data smoothness and volatility. First, we model the time series of ECG signals as a graph with multiple nodes, each connected to two neighbors (except the ends). Then selecting α, α value is swept from −∞ to + ∞, to identify troughs and match them to peaks as it increases. When α is swept passed a node that it identifies as a local minimum, it saves the value of that node as the birth of that trough. The death of a trough is given by the lowest α value. Finally, the algorithm terminates when all node values are smaller than α. We select two acceptable ECG signals and two unacceptable ECG signals and use the SLS filtration method to generate persistence diagram, as shown in Figure 5C below.

### Evaluation method

We select the following evaluation indicators to obtain the classification accuracy: sensitivity (*Se*), specificity (*Sp*), *F*1, accuracy (*Acc*) and correction accuracy (*mAcc*), which are defined as follows:

*Se*: The number predicted to be positive and correct, the proportion of the total number of actual positives.


(4) $$S\,e=\frac{\text{TP}}{\left(\mathrm{TP}+\mathrm{FN}\right)}\times 100\%$$

*Sp*: The number predicted to be negative and correct, the proportion of the total number of actual negatives.


(5) $$S\,p=\frac{\text{TN}}{\left(\mathrm{TN}+\mathrm{FP}\right)}\times 100\%$$

*F*1: The harmonic values of the precision rate and recall rate.


(6) $$F\,1=\frac{\text{TP}}{\mathrm{TP}+0.5\,\left(\mathrm{FR}+\mathrm{FN}\right)}\times 100\%$$

*Acc*: Number of correct predictions, accounting for the total number.


(7) $$A\,c\,c=\frac{\mathrm{TP}+\mathrm{TN}}{\mathrm{TP}+\mathrm{TN}+\mathrm{FR}+\mathrm{FN}}\times 100\%$$

*mAcc*


(8) $$m\,A\,c\,c=\frac{\left(S\,e+S\,p\right)}{2}\times 100\%$$

Among them, TP: “acceptable” signal is correctly predicted as an “acceptable” signal by the model; TN: “unacceptable” signal is correctly predicted as an “unacceptable” signal by the model; FP: “unacceptable” signal is incorrectly predicted as an “acceptable” signal by the model; FN: “acceptable” signal is predicted as an “unacceptable” signal by the model’s signal.

## Results

In this study, a quality assessment method of wearable dynamic ECG signals was proposed based on the persistent homology method and GoogLeNet (Assari et al., 2022) method. The performances of VR and SLS filtration were considered. We put the 224 × 224 × 3 persistent barcode pictures or persistent diagram pictures obtained by the persistent homology method into GoogLeNet for classification. For VR filtration, the influences of the sliding windows length for the point cloud structure was also explored. Tenfold cross-validation is applied to test classification performance. All the segments were randomly divided into 10 groups.

### Results of 12-leads ECGs dataset

**FIGURE 6:**
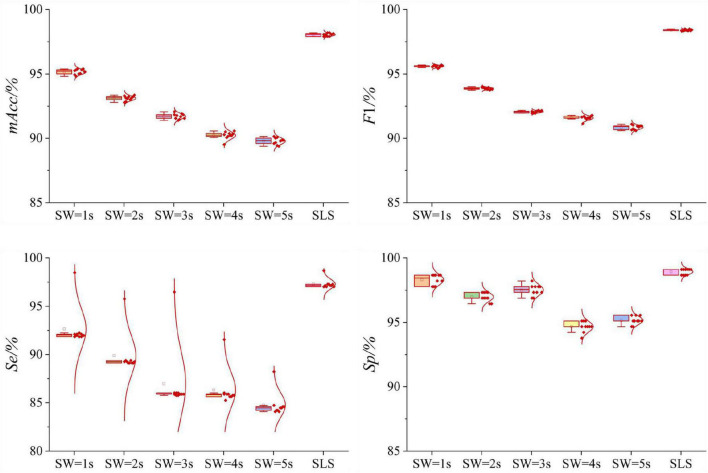
Boxplots of the normal distribution curves of the 10-fold cross-validation of VR and SLS filtration methods in 12-leads ECGs Dataset. For the VR method, when the length of sliding window increases gradually, the average value of *mAcc* decreases gradually. The 10-fold cross-validation results of *mAcc*, *F*1, *Se*, *Sp* are relatively concentrated. For the SLS method, the results of 10-fold cross-validation of *mAcc*, *F*1, *Se*, *Sp* are relatively scattered. But the average value of *mAcc* is higher than that of VR method.

Table 1 and Figure 6 displayed the results of 12-leads ECGs Dataset. For VR filtration, the length of sliding windows was set to 1, 2, 3, 4, and 5 s, respectively. As shown in Table 1, the classification result of SLS filtration method is the best, *mAcc* = 98.04%. The result of VR filtration method with sliding window of 1 s is the relatively high, *mAcc* = 95.16%. As the length of sliding windows increasing, the classifying performances decrease. In the Figure 6, the Box-plot and normal distribution curve of all results were given. The boxplot shows the mean and variance of 10-fold cross-validation results, while the normal distribution curve shows how the results distribute.

**TABLE 1:** Classification results of VR and SLS filtration methods in 12-leads ECGs Dataset.

	SW = 1 s	SW = 2 s	SW = 3 s	SW = 4 s	SW = 5 s	SLS
*mAcc* (%)	95.16 ± 0.20	93.11 ± 0.19	91.75 ± 0.21	90.22 ± 0.29	89.78 ± 0.26	**98.04 ± 0.11**
*F*1 (%)	95.60 ± 0.09	93.88 ± 0.08	92.06 ± 0.08	91.58 ± 0.18	90.85 ± 0.17	**98.40 ± 0.06**
*Se* (%)	92.02 ± 0.14	89.25 ± 0.35	85.90 ± 0.11	85.78 ± 0.23	84.41 ± 0.24	**97.15 ± 0.11**
*Sp* (%)	98.31 ± 0.41	96.98 ± 0.42	97.51 ± 0.43	94.67 ± 0.42	95.16 ± 0.33	**98.93 ± 0.23**

Bold values represent the data with the best results.

### Results of single-lead ECGs dataset

**FIGURE 7:**
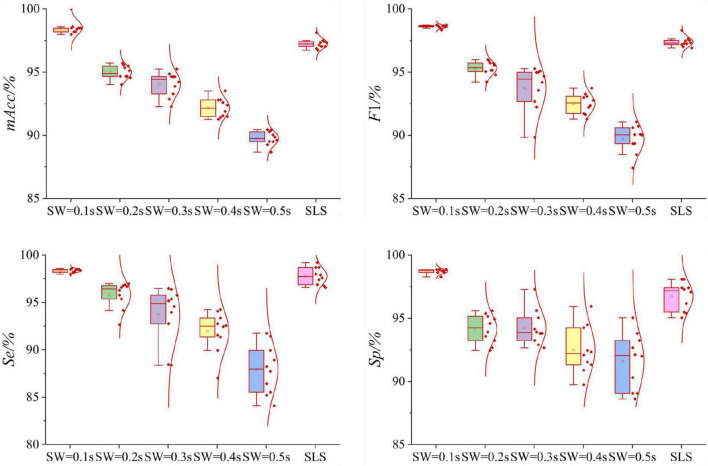
Boxplots of the normal distribution curves of the 10-fold cross-validation of VR and SLS filtration methods in single-lead ECGs Dataset. For the VR method, when the length of sliding window increases gradually, the average value of *mAcc* decreases gradually. The 10-fold cross-validation results of *mAcc* with different sliding windows are relatively concentrated, and the results of *F*1, *Se*, *Sp* are relatively scattered. For the SLS method, the 10-fold cross-validation results of *mAcc* is relatively concentrated, while the results of *F*1, *Se*, *Sp* are relatively scattered. But the average value of *mAcc* is slightly lower than that of VR method.

Table 2 and Figure 7 displayed the results of single-lead ECGs Dataset. For VR filtration, the point cloud structure of sliding windows with five lengths of 0.1, 0.2, 0.3, 0.4, and 0.5 s were established, respectively. In the Figure 7, the Box-plot and normal distribution curve of all results were also given. As shown in Table 2, the experimental results for the VR filtration show that the *mAcc* of the point cloud dataset with a sliding window length of 0.1 s is 98.55%, and the standard deviation is 0.13%. As the length of sliding windows increasing, the classifying performances decrease. The *mAcc* of the persistence diagram obtained by SLS filtration is 97.25%, and the standard deviation is 0.39%.

**TABLE 2:** Classification results of VR and SLS filtration methods in single-lead ECGs Dataset.

	SW = 0.1 s	SW = 0.2 s	SW = 0.3 s	SW = 0.4 s	SW = 0.5 s	SLS
*mAcc* (%)	**98.55 ± 0.13**	94.98 ± 0.55	94.06 ± 0.96	92.17 ± 0.76	89.76 ± 0.56	97.25 ± 0.39
*F*1 (%)	**98.62 ± 0.12**	95.32 ± 0.56	93.75 ± 1.73	92.51 ± 0.79	89.71 ± 1.11	97.39 ± 0.39
*Se* (%)	**98.37 ± 0.18**	95.81 ± 1.41	93.73 ± 3.13	91.93 ± 2.16	87.93 ± 2.63	97.77 ± 0.89
*Sp* (%)	**98.85 ± 0.20**	94.15 ± 1.14	94.24 ± 1.40	92.50 ± 1.87	91.59 ± 2.22	96.74 ± 1.13

Bold values represent the data with the best results.

## Discussion

This study proposed a new signals quality assessment method of wearable dynamic ECGs based on persistent homology method and GoogLeNet method. This method has strong robustness in quality assessing, which can be used for both 12-leads and single-lead ECG signals. VR and SLS two filtration methods were employed for persistent homology feature extraction. For the 12-leads ECGs Dataset, SLS filtration method has the best classification performance, *mAcc* = 98.04%, while for the single-lead ECGs Dataset, the classification result of VR filtration method with sliding window of 0.1 s is the highest, *mAcc* = 98.55%.

### Comparison between VR filtation and SLS filtation

**FIGURE 8:**
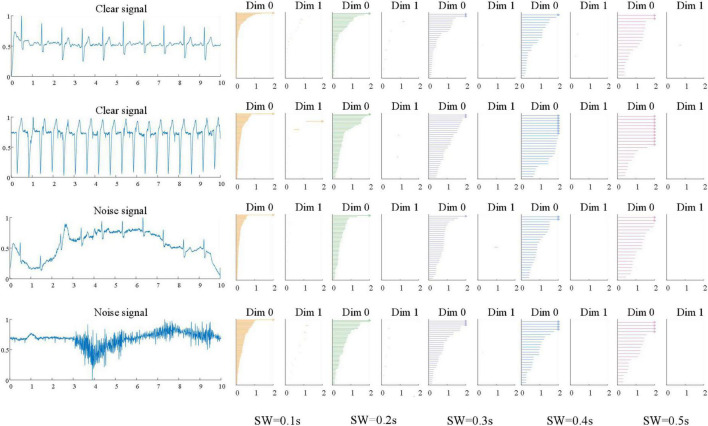
Persistence barcode pictures of “acceptable” and “unacceptable” ECG signals with five sliding window lengths of single-lead ECGs Dataset.

In this study, VR filtration method needs to reconstruct the time series of ECG signal, while sliding window method is used to construct the point cloud structure of ECG signal. For 12-leads ECGs Dataset, the sliding window lengths are 1, 2, 3, 4, and 5 s, respectively, and the dimensions are 120, 60, 36, 24, and 24, respectively. The results show that the sliding window is 1 s, and the classification result is the highest. For the single-lead ECGs Dataset, the sliding window lengths are 0.1, 0.2, 0.3, 0.4, and 0.5 s, respectively, and the dimensions are 100, 50, 33, 25, and 20, respectively. It can be seen that the classification accuracy with a sliding window length of 0.1 s is the highest, the average *mAcc* of the 10-fold cross-validation is as high as 98.55%. We found that for the two datasets, the classification accuracy decreases with the increase of window length. In this study, the sliding window length will not continue to decrease in the two datasets. In the 12-leads ECGs Dataset, for 12 leads signals, the further decrease of sliding window will cause the data dimension to be too large, which will increase the calculation cost. In the single-lead ECGs Dataset, the sliding window length does not continue to shrink because the classification accuracy achieves good results when the window length is 0.1 s. Taking the single-lead ECGs Dataset as an example, the “acceptable” and “unacceptable” ECG signal waveforms and persistence barcode of the five sliding window lengths are shown in Figure 8. It can be seen from the figure that the persistent barcodes of ECG signals with different window sizes are obviously different. As the window increases, the persistent barcodes gradually become sparse. There are also differences between “acceptable” and “unacceptable” ECG signals corresponding to persistent barcode.

In this study, in the 12-leads ECGs Dataset, the classification accuracy using the SLS filtration method is higher than the highest accuracy using the VR filtration method; in the single-lead ECGs Dataset, the classification accuracy using the SLS filtration method is lower than the highest accuracy using the VR filtration method. SLS filtration method finds the birth and death points on the waveform of the ECG signal. The VR filtration method uses a simple complex to reconstruct geometry to analyze the spatial characteristics of the ECG signal time series. However, VR filtration method depends on the construction of point cloud. SLS filtration method is more stable. In the 12-leads ECGs Dataset, *mAcc* = 98.04% and in the single-lead ECGs Dataset, *mAcc* = 97.25%. From the classification results, we can see that the classification results of the two datasets based on this method are relatively good.

### Comparison with other methods

In this study, SLS method is compared with some ECG signal quality evaluation methods in recent years, and the results are shown in Table 3. For example, Zhao and Zhang (2018) proposed a simple heuristic fusion and fuzzy comprehensive evaluation method based on SQI for ECG quality evaluation, with an accuracy rate of 94.67% on the test set. Jin et al. (2022) proposed a novel dual attention convolution long short-term memory neural network for ECG quality assessment, and the final classification accuracy was 94%. Shahriari et al. (2017) developed an image based ECG quality assessment technique with an accuracy of 82.50%; Zhang et al. (2019) conducted performance testing in ECG quality assessment by comparing seven feature schemes composed of random forest, SVM and its variants combined with nonlinear features, among which least squares SVM had the highest Acc of 92.20% in test data. Because the dataset is unbalanced, according to formula (8), we choose the *mAcc* to calculate the classification results. In order to unify the evaluation standard with other studies, we also calculate the *Acc* according to formula (7).

**TABLE 3:** Performance of the presented algorithm and methods participating in the PhysioNet/CinC Challenge.

	*mAcc* (%)	*Acc* (%)	*Se* (%)	*Sp* (%)
Kalkstein et al. (2011)	86.30	93.00	74.10	98.50
Xia et al. (2011)	89.11	85.90	95.11	83.10
Zhao and Zhang (2018)	91.67	94.67	90.33	93.00
Jin et al. (2022)	87.03	94.00	97.59	76.47
Shahriari et al. (2017)	80.80	82.50	83.90	77.70
Zhang et al. (2019)	88.02	92.20	77.94	98.09
Proposed SLS method	**98.04**	**97.70**	**97.15**	**98.93**

*Se, Sp, Acc* and *mAcc* for the 12-leads ECGs Dataset. Bold values represent the data with the best results.

**FIGURE 9:**
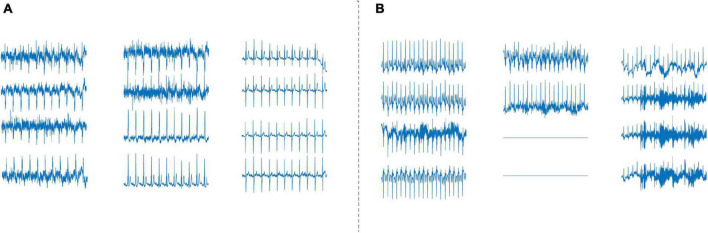
**(A)** The “acceptable” 12-leads ECG signal, **(B)** The “unacceptable” 12-leads ECG signal.

As can be seen from Table 3, the classification result of SLS filtration method is still the highest in the 12-leads ECGs Dataset. However, the labeling for “acceptable” or “unacceptable” for the whole 12 channels was not clear. In a 12-leads ECG signal, some single-lead signals are “acceptable” and some single-lead signals are “unacceptable.” As shown in the Figure 9. We selected an “acceptable” and an unaccep2-leads ECG signal. From (A), we can see that there is also obvious noise in the “acceptable” 12-leads ECGs. From (B), We can see that in the “unacceptable” 12-leads ECG signal, the single-lead ECG signal may be bad, but part of the heart rate information could be calculate. Therefore, we considered the quality evaluation of single-lead ECG signals.

For single-lead ECGs Dataset, Table 4 shows the comparison between VR filtration method and the other four methods. The references are as follows: reference (Liu et al., 2020) selected 26 signal quality indicators (SQI) to evaluate the quality of ECG signals, including time domain characteristics, frequency domain characteristics, and SQI based on QRS wave and nonlinear characteristics. Experiments were conducted to test the performance of a single classifier based on SQI features and multiple classifiers based on SQI features. The total classification performance of a single classifier based on GbSQI features and the total classification performance of multiple classifiers based on GbSQI features are obtained. For the overall classification performance of a single GbSQI-based classifier, the QRS wave-based SQIs had the best performance; for example, the *mAcc* of bSQI_2 was (93.84 ± 1.47)% and bSQI_4 *mAcc* was (93.70 ± 1.49)%. For the overall classification performance of multiple classifiers based on GbSQI features, the effect of the classification model was best when 14 SQIs were selected, and the *mAcc* was 95.2%. Reference (Liu et al., 2018) redefined the bSQI with any two combined QRS detectors and then extended the redefined bSQI to the bSQI of multiple QRS detectors, represented by GbSQI. The experimental results showed that the mAcc of the classifier with the best combination of six QRS detectors and a single GbSQI feature was 94.03%. The best combination of four QRS detectors was the GbSQI feature, and the *mAcc* of multiple classifiers was 94.76%. Study (Falk and Maier, 2014) proposed a quality index of ECG signals based on modulation spectrum signal representation, and a quality index MS-QI based on modulation spectrum was proposed. The experimental results of the above comparison methods are based on the original challenge data. In order to compare the performance of the methods proposed in this study, we selected several commonly used indicators from the reference literature to test based on the single-lead ECG datasets built in this study. A comparison of the results is shown in Table 4. According to the table, the classification accuracy of the VR filtration method is still the highest.

**TABLE 4:** Comparison of classification accuracy results of single-lead ECGs Dataset four methods.

Methods	*mAcc* (%)	*F*1 (%)	*Se* (%)	*Sp* (%)
MS-QI Falk and Maier (2014)	85.61 ± 1.05	85.58 ± 0.48	81.57 ± 0.73	89.66 ± 1.77
bSQI_2 Liu et al. (2018)	84.08 ± 0.75	84.85 ± 0.33	83.79 ± 0.71	84.37 ± 1.90
bSQI_4 Liu et al. (2018)	89.28 ± 0.55	89.33 ± 1.07	86.21 ± 1.49	92.36 ± 1.20
picaSQI Liu et al. (2020)	93.11 ± 0.73	93.03 ± 0.58	89.33 ± 1.11	96.90 ± 0.58
Proposed VR method	**98.55 ± 0.13**	**98.62 ± 0.12**	**98.37 ± 0.18**	**98.85 ± 0.20**

Bold values represent the data with the best results.

In this study, the wearable ECG signals were graded into two groups: “acceptable” vs. “unacceptable.” However, part of wearable ECG signals only R wave could be detected, other waves like P or ST were drowned out by the noise, as shown in Figure 9. These signals cannot be used for some CVDs detection, but they also cannot be abandoned as heart rate information can be obtained. Therefore, it is not appropriate to simply divide ECG signals into acceptable and unacceptable. A more detailed quality evaluation grades need to be considered in the future.

## Conclusion

Cardiovascular disease poses a threat to human health, with tens of millions of deaths worldwide every year. Its prevention and monitoring are urgent issues. This study proposed a new signals quality assessment method of wearable dynamic ECGs based on persistent homology method and GoogLeNet method. This method has strong robustness in quality assessing, which can be used for both 12-leads and single-lead ECG signals. VR and SLS two filtration methods were employed for persistent homology feature extraction. The VR filtation method selected persistence barcode to quantify the topological features, and the SLS filtation method selected persistence diagram to quantify the topological features. When using VR filtation method, it is necessary to reconstruct the time series. The focus of this method is to use the sliding window method to construct the point cloud dataset. For 12-leads ECGs Dataset, the sliding window sizes are 0, 1, 2, 3, 4, and 5 s, respectively, and 120, 60, 36, 24, and 24 dimensions are established, respectively. When use the SLS filtation method, the classification result of 12-leads ECGs Dataset is the highest, *mAcc* = 98.04%. For single-lead ECGs Dataset, the sliding window sizes are 0.1, 0.2, 0.3, 0.4, and 0.5 s, respectively, and 100, 50, 33, 25, and 20 dimensions are established, respectively. The classification results show that when the window length of VR filtration method is 0.1 s, the classification result is the highest, *mAcc* = 98.55%. The results show that persistence homology method performed well in the quality evaluation of wearable ambulatory ECG. This study verified the feasibility of applying the persistence homology method to wearable ECG signal quality assessment. In this study, the persistent homology method is still insufficient. We need the sliding window method to find the optimal point cloud matrix. In the next experiment, we integrate the experiment and the method to find an optimal point cloud construction method. This study is to classify acceptable and unacceptable ECG signals. In the next work, we will continue to refine the classification criteria to make Wearable ECG instruments more widely used.

## Data availability statement

Publicly available datasets were analyzed in this study. This data can be found here: https://physionet.org/content/challenge-2011/1.0.0/.

## Author contributions

YR, FL, and SX designed the research. YR designed the algorithm, analyzed the data, and wrote the manuscript. FL contributed to the signal relabeling. SS, LC, and ZW contributed to the collecting and sorting data. FL and SX contributed to the revising it critically for the important intellectual content. All authors contributed to the article and approved the submitted version.
